# Development of *p*-*y* Curves of Laterally Loaded Piles in Cohesionless Soil

**DOI:** 10.1155/2014/917174

**Published:** 2014-01-16

**Authors:** Mahdy Khari, Khairul Anuar Kassim, Azlan Adnan

**Affiliations:** Department of Geotechnics and Transportation, Faculty of Civil Engineering, Universiti Teknologi Malaysia, 81300 Skudai, Johor Bahru, Malaysia

## Abstract

The research on damages of structures that are supported by deep foundations has been quite intensive in the past decade. Kinematic interaction in soil-pile interaction is evaluated based on the *p*-*y* curve approach. Existing *p*-*y* curves have considered the effects of relative density on soil-pile interaction in sandy soil. The roughness influence of the surface wall pile on *p*-*y* curves has not been emphasized sufficiently. The presented study was performed to develop a series of *p*-*y* curves for single piles through comprehensive experimental investigations. Modification factors were studied, namely, the effects of relative density and roughness of the wall surface of pile. The model tests were subjected to lateral load in Johor Bahru sand. The new *p*-*y* curves were evaluated based on the experimental data and were compared to the existing *p*-*y* curves. The soil-pile reaction for various relative density (from 30% to 75%) was increased in the range of 40–95% for a smooth pile at a small displacement and 90% at a large displacement. For rough pile, the ratio of dense to loose relative density soil-pile reaction was from 2.0 to 3.0 at a small to large displacement. Direct comparison of the developed *p*-*y* curve shows significant differences in the magnitude and shapes with the existing load-transfer curves. Good comparison with the experimental and design studies demonstrates the multidisciplinary applications of the present method.

## 1. Introduction

Significant damages of structures supported by deep foundations due to complete or partial collapse during earthquakes has been observed in the past (El Naggar and Novak., 1995; [1–3]). These authors have demonstrated the paramount importance of the soil-pile-superstructure interaction (SPSI) in the seismic behavior of structures [4]. Kinematic interaction in SPSI is due to presence of pile foundation in the ground. Several methods are widely used to determine the kinematic interaction, such as finite element method (FEM) [5, 6], boundary element method (BEM) [1, 7], and beam on nonlinear winkler foundation (BNWF) [2, 4, 8, 9]. The FEM and BEM approaches are versatile techniques. Although, the SPSI analysis can be performed coupled without site response analysis, it is very expensive from the calculation viewpoint [4]. To evaluate the behavior of laterally loaded pile in the kinematic interaction, the BNWF method is widely used in research practices [4, 9–11]. McClelland and Focht [12] organized the BNWF method. In the BNWF method, the soil and pile are modeled as nonlinear springs and linear elements, respectively. The stiffness coefficient of the spring is evaluated based on the load-transfer approach, often known as *p*-*y* curve method.

Many investigators have developed *p*-*y* curves for clayey soils [10, 13, 14] and for sandy soils [10, 15, 16]. However, these developed curves do not account some parameters such as relative density of sandy soil, side friction, and bending stiffness of the pile. This research has aimed to develop a series of *p*-*y* curves through comprehensive experimental investigations in Johor Bahru sand for a single pile subjected to lateral load.

## 2. Brief Review

The behavior of soil-pile interaction has been analyzed using the concept of subgrade modulus. In fact, the static equilibrium between pile and surrounding soil must be initially obtained. Kondner [17] performed a series triaxial compression tests to obtain the stress-strain relationship of soil where the *p*-*y* curves by hyperbolic function were established. In 1970, Matlock assumed that soil reaction at a point dependent only on the pile deflection at that point and the reaction is independence of pile deflection in the depths of above and below of the point of interest. Reese et al. [15] developed a *p*-*y* curve from the of the full scale load tests. The curve consisted of an initial straight line (*p* = *k*_*py*_ *zy*; *z* = desired depth; *k*_*py*_ = subgrade modulus), a parabolic section ($p=\hat{C}y^{1/n}$; *C* = *p*_*m*_/*y*_*b*_^1/*n*^; *p*_*m*_ is soil pressure at *y*_*b*_) between *y*_*b*_ = *D*/60 and *y*_*u*_ = 3*D*/80 (*D* = pile diameter); and a final straight line (*p* = *A*_*s*_ *p*_*u*_; *A*_*s*_ = empirical factor; *p*_*u*_ = ultimate resistance derivation from analysis of a wedge) established at *y*_*u*_. It is noteworthy that the curve is same for sands in both above and below the water table. Weeselink et al. [18] developed *p*-*y* curves for use in calcareous soils which is given below as follows:

(1) $$\begin{array}{c} P=R{\left(\frac{z}{z_{0}}\right)}^{n}{\left(\frac{y}{D}\right)}^{m}, \end{array}$$

where *z*_0_ = constant length taken as 1 m; *R* = control variable for curve stiffness (850 kPa); and *m* and *n* are empirical factors. Other researchers developed the values of *R*, *n*, and *m* [19]. In 2001, Dyson and Randolph developed the curves for use in calcareous soils. They added the unit weight factor to Weeselink's equation. Scott [20] performed centrifuge tests to investigate the soil-pile interaction and suggested the bilinear function to model the *p*-*y* curve as presented in the following equation:

(2) $$\begin{array}{c} P=\frac{{D\sigma }_{0}^{\prime }}{{\left(\text{1}/\pi \right)\left(1/\sin^{2}Q\;+1/(3-4D)\right)}^{0.5}}, \end{array}$$

where *σ*_0_′ = (*σ*_1_′ + *σ*_2_′ + *σ*_3_′)/3.

O'Neill and Murchison (1984) studied the *p*-*y* curves in sand and modified the suggested equations by Reese et al. [15] as shown in the following equation:

(3) $$\begin{array}{c} P=\eta AP_{u}\tanh\left[\frac{kzy}{AP_{u}}\right], \end{array}$$

where *η* and *k* are factors for shape of pile (i.e., for circular cross-section = 1) and initial modulus of subgrade reaction, respectively; *D* is pile diameter and *z* is the depth; *A* is a coefficient based on loading conditions (static or cyclic); and *P*_*u*_ is determined based on wedge type and flow failure which takes from the smaller from the following equations:

(4) $$\begin{array}{c} P_{ud}=C_{3}Dzy,\;\;P_{su}=\left(C_{1}z+C_{2}D\right)z\gamma . \end{array}$$

The three coefficients *C*_1_, *C*_2_, and *C*_3_ (as function of the friction angle) are used to calculate the ultimate soil resistance. The initial modulus of soil reaction is computed using the experimental factor *k*. This method is adopted by the API [21] as a modified shape of the *p*-*y* curves for sand for offshore pile foundations.

## 3. Experimental Work

**Figure 1:**
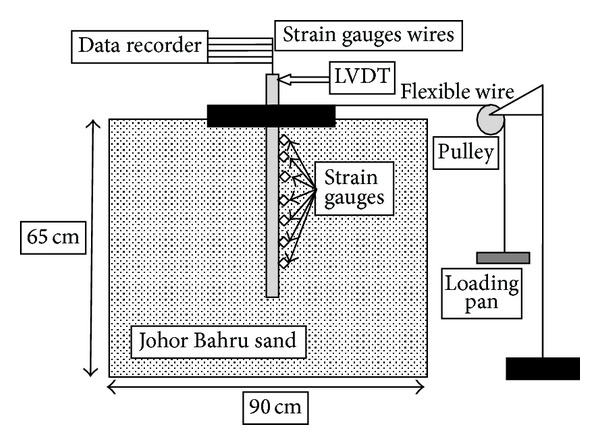
Schematic view of experimental setup.

The schematic diagram of the test setup is shown in Figure 1. The dimensions of rectangular soil tank were 900 mm in length, 700 mm in width, and 65 mm in height. To minimize the box boundaries effects, the size of soil tank was extended up to 8–12*D* and 3-4*D* in the direction and perpendicular to lateral load, respectively [22]. In additional, the soil thickness was kept below pile tip at least 6*D*.

Lateral load was applied to piles at the surface of the model ground through a pulley arrangement with flexible wire attached to pile cap (Figure 1). The other end was attached to the loading pan. The loads were applied by gradually increasing the dead weight in the pan.

### 3.1. Model Pile and Instrumentation

**Figure 2:**
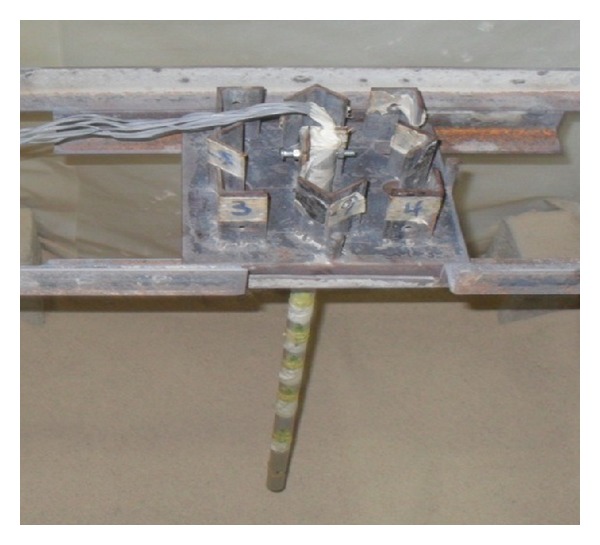
Pile and pile cap setup in soil box.

As Figure 2 shows, the model pile was made of aluminum alloy tubing with a value of Young's modulus of 69.8 GPa, 15.88 mm in out diameter (*D*), and 13.88 mm in inside diameter. The embedded length-to-diameter ratio (*l*/*D*) of pile was equal to 32. The pile diameter was around 16 times greater than the maximum particle size for sand which satisfied the recommended ratios in excess of 15–30 to avoid scale effects [23]. The properties of the model pile were scaled by a dimensional analysis (Buckingham Pi theorem) with the properties of the basic prototype pile of Penang Second Crossing in Malaysia. A steel plate was used as a pile cap for the single pile. To satisfy the fixed head conditions, the pile was passed through a hole in the cap and then screwed to angle profiles (length = 50 mm) welded on the hole (Figure 2). The tests were conducted on smooth (wall friction of pile was low) and rough (fine sand was pasted around the pile by adhesive), single piles in dense (*D*_*r*_ = 75%), and loose (*D*_*r*_ = 30%) sand.

A typical test (shown in Figure 1) included nine instruments: two linear variable differential transducer (LVDT) to measure the deflection of the pile and seven levels of electrical strain gauges having resistance of 350 ± 0.1 *Ω* to measure bending moments. The strain gauges were fixed along the outer surface of the pile. The distances of the gauges were at closer and larger spacing near ground surface and towards the pile tip, respectively (Figure 2). They were coated with a 0.5 mm thick layer of epoxy for protection.

**Figure 3:**
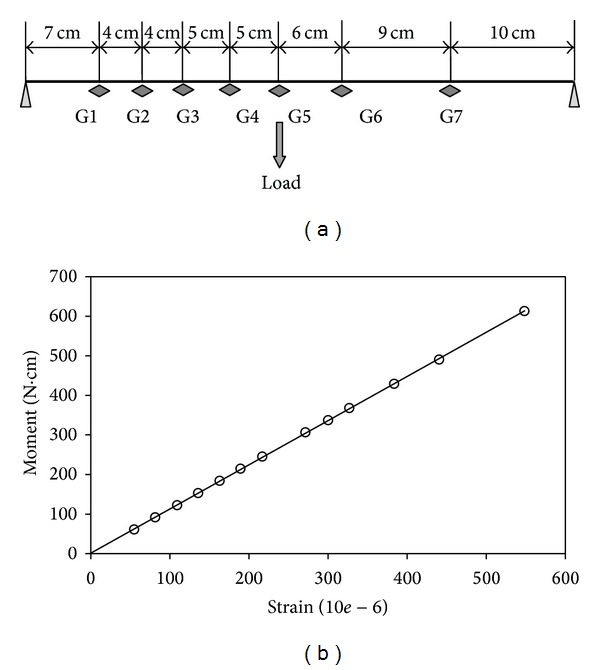
Relationship between strain and moment. (a) Pile in pure moment; (b) fitted straight line.

Gauge constants were calculated for every one of the gauges separately. The pile was supported on a set of edges. Fifteen different pure moments were applied over the central portion of pile (Figure 2). The values of observed strain and applied moments were correlated to compute the gauge constant at each gauge location (Figure 3). The constant was determined by means of the method of least squares. During the calibration, the central deflection of pile was observed. The flexural stiffness (EI) was computed to be 91 × 10^6^ N·mm^2^.

### 3.2. Soil Properties and Sample Preparation

**Figure 4:**
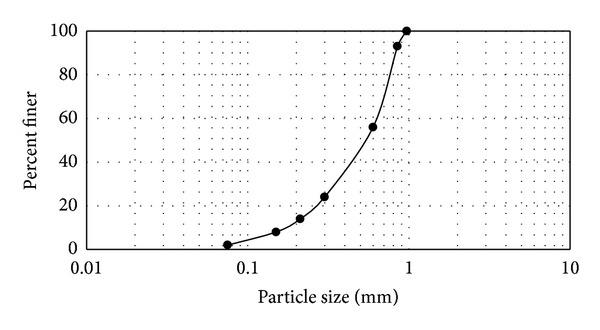
Gradation curve of the Johor Bahru sand.

The tests conducted on the dried sand (i.e., in the laboratory temperature). The soil samples were from Johor Bahru in Malaysia. The sampled sand was classified as *SP,* according to the Unified Soil Classification System (*USCS*). The medium diameter (*D*_50_) and uniformity coefficient (*C*_*u*_) of sand were 0.532 and 0.17 mm, respectively; and particle sizes in a range of 0.075–0.97 mm with the gradation are shown in Figure 4. Based on British Standard methods (BS-1377), minimum and maximum unit weights of sand were 13.74 kN/m^3^ and 16.38 kN/m^3^.

**Figure 5:**
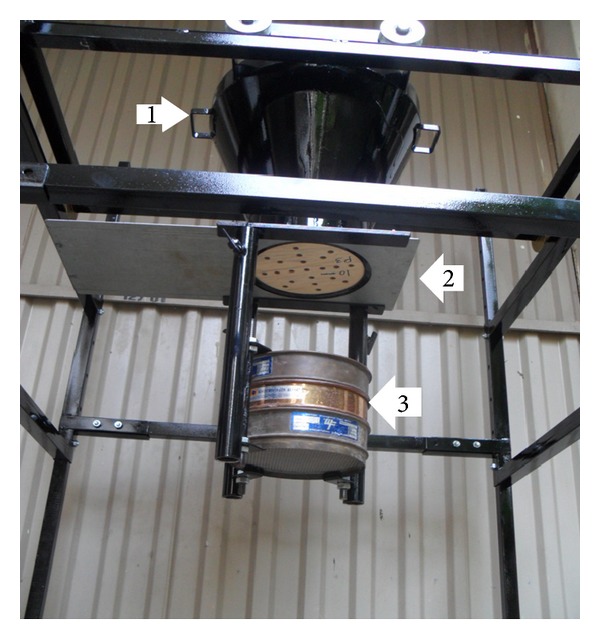
Mobile Pluviator System.

To reconstruct the sand samples, several methods have been developed by investigators such as vibration, tamping, and pluviation [24]. The prepared samples using the pluviation and tamping technique often result in a specimen of homogenous and nonuniform density, respectively. Accordingly, the newly designed mobile pluviator was utilized in this research to reconstruct the dry sandy soil samples using the dry pluviation method (Figure 5). The newly developed mobile pluviator by Khari et al. [11] consisted mainly of a soil bin (hopper, Figure 5, no. 1), the diffuser system (the three sieves, Figure 5, no. 3), and sand collector, a fixing device to set up these components so as the whole system was carried by a moveable steel frame. As Figure 5 shows, the interchangeable circular wood plates (shutter plates, Figure 5, no. 2) were installed in the bottom of the sand hopper. The four patterns of the shutter plates were formed in a different manner of the distribution of the holes for the sake of controlling the rate of the soil discharge. While the apparatus was movable, the different factors were examined to obtain a wide range of the relative densities. The falling height and the rate of pouring had the opposite effects on the relative density [25]. Based on the obtained results, the two patterns selected consisted of 11 holes (diameter = 18 mm) and 16 holes (diameter = 10 mm) distributed evenly in the shutter to achieve the dense and the loose sand samples with relative density of 75% and 30%, respectively. The falling height was kept constant at 700 mm from the surface of the model ground, so it was more than the critical height to obtain terminal velocity. The pour was stopped when the height of sand rained in the soil tank was 30 mm thicker than the required height and finally the extra soils were removed.

### 3.3. Test Procedure

The piles were first located in the center of the soil tank and fixed with the cap. Verticality of the pile was maintained using a guide frame. After placing the model pile, the soil box was filled with the dried sand using the mobile pluviator apparatus.

To monitor uniformity and relative density during the samples preparation, three small boxes (cylinder shaped, with a volume of 455 cm^3^) were placed on the surface of sample prior to sand spreading. The surface of the model ground was leveled when the required height was achieved. At least 24 hours elapsed before applying any load to the pile. To eliminate any time effects due to sand consolidation, strain gauge readings were taken after 10 minutes for each load increment. The data measured from the LVDTs and strain gauges were stored on a computer data acquisition system.

## 4. Results and Discussion

A series of tests were performed on single fixed-head piles in loose (*D*_*r*_ = 30%) and dense (*D*_*r*_ = 75%) sand. The tests T44 and T45 were performed on smooth piles; and T48 and T47 were conducted on rough piles in dense and loose sand, respectively. The loads were applied to piles in an incremental manner.

The strain values obtained were converted to moments by multiplying strain by the previously estimated gauge constants. A smooth fourth-order polynomial was then fitted through the experimentally moments observed. Lateral displacements (*y*) and soil-pile reactions (*p*) were computed by double integration and differentiation of moment curve along the depth of pile, respectively.

**Figure 6:**
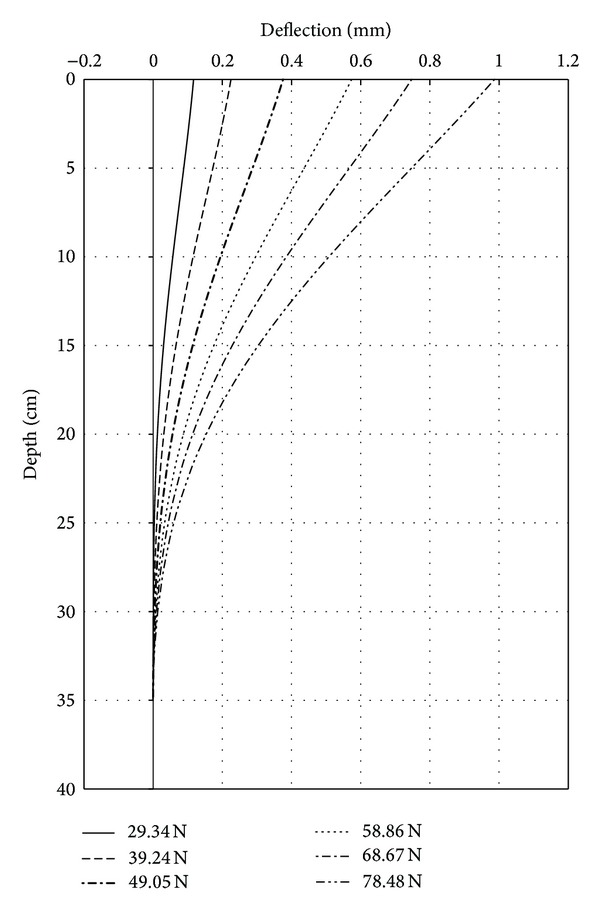
Deflection versus depth test 44.

The variation of deflection (*y*) along pile is presented in Figure 6. As stated earlier, the integration of the slope curve leads to deflection curves versus depth along the pile. It can be noted that, at a depth of 26 to 28 cm, pile did not show any deflection because of active length of pile (Figure 6).

**Figure 7:**
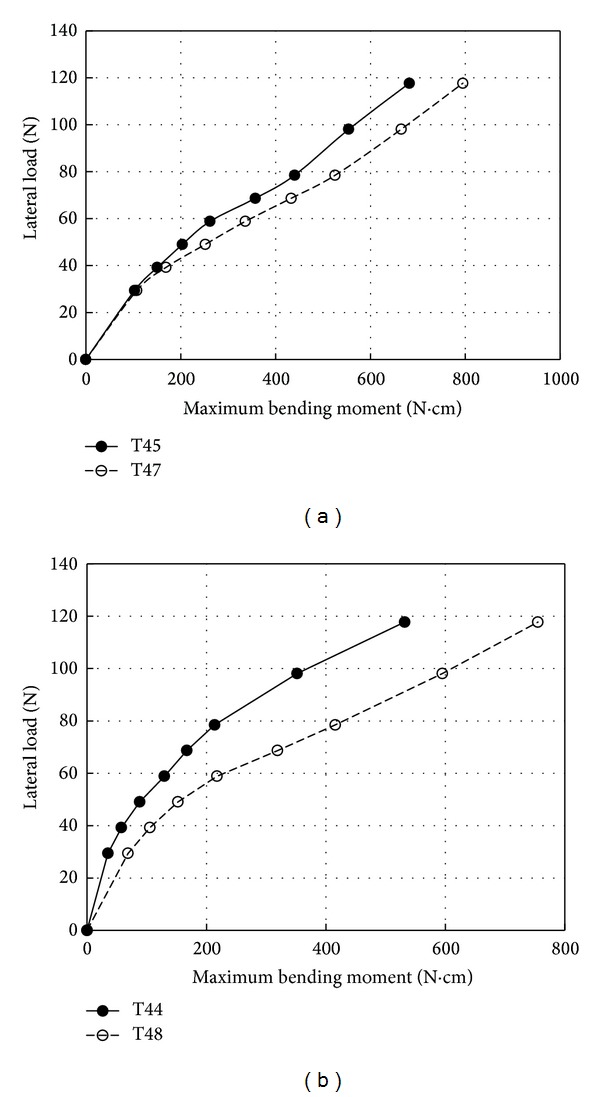
Maximum bending moment versus lateral load; (a) *D*_*r*_ = 30%; (b) *D*_*r*_ = 75%.

**Figure 8:**
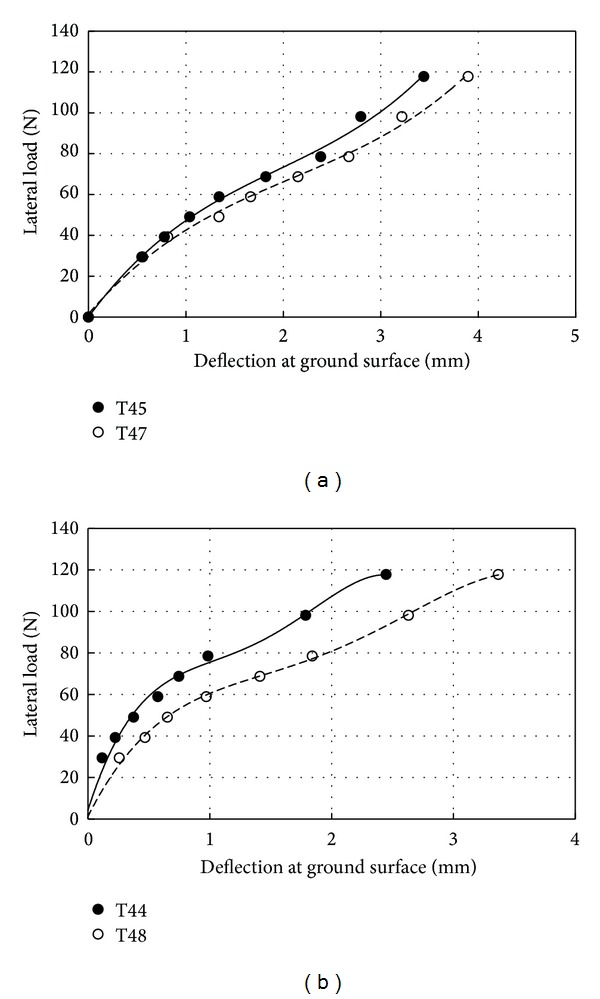
Deflection at ground surface versus lateral load; (a) *D*_*r*_ = 30%; (b) *D*_*r*_ = 75%.

Figure 7 shows the effects of relative density on the maximum bending moment along the pile. As can be seen in Figure 8(b), the maximum bending moments were observed in the rough pile in loose and dense densities. This is primarily due to the fact that as friction between soil and pile increased its impact on the soil-pile reaction was higher. This result can be derived from Figure 6 as well. However, a comparison of the results shown in Figures 7(a) and 7(b) explains the effect of the roughness of the wall pile is more significant in soil with the higher relative density. Thus, the maximum bending moments were increased about 75% and 24% for the relative density of *D*_*r*_ = 70% and 35%, respectively.  Figure 8 illustrates the differences in deflection at ground surface against applied lateral loadings for smooth and rough pile in the loose and dense sand. The deflections measured using LVDTs and those obtained from the integration process (Lines in Figure 8) were in good agreement. The results indicate that the deflection of the smooth pile located in loose sand was 200% larger than the embedded pile in the dense sand. The value of the deflection was increased in rough pile about 175% and 23% for the *D*_*r*_ = 75% and *D*_*r*_ = 30%, respectively, compared to the displacement occurred in smooth pile.

As stated, to evaluate the load-transfer (*p*-*y*) curve, the bending moments at each gauge station were computed with multiplying the strains recorded by the gauge constant measured. The experimental bending moment data attained were fitted with smooth fourth-order polynomial. The bending moment curvature *M*(*z*) was then double differentiated and integrated to obtain soil-pile reaction (*p*) and lateral pile deflection (*y*) as presented in the following equations respectively:

(5) $$\begin{array}{c} p=\frac{d^{2}M\left(z\right)}{d^{2}z}, \end{array}$$

(6) $$\begin{array}{c} y=\iint \frac{M\left(z\right)}{E_{P}I_{P}}\;dz. \end{array}$$

The integration constant was deduced by matching the measured rotation and deflection at pile head. As shown in (7), the *p*-*y* curves were obtained by combining the ultimate soil-pile reaction (*p*_*u*_) and the initial horizontal subgrade modulus (*k*_ini_) of *p*-*y* curve to produce curves for each depth, fitted by a hyperbolic relationship of the form:

(7) $$\begin{array}{c} p=\frac{y}{1/k_{\text{ini}}+y/p_{u}}. \end{array}$$

**Figure 9:**
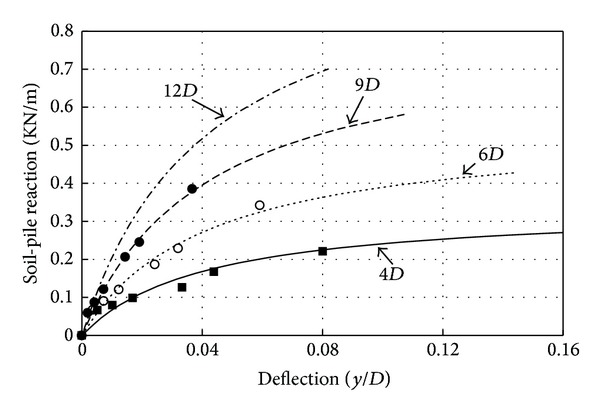
Typical fitted and experimental load-deflection curves in different depth for Johor Bahru sand. *Note*. (OB: observed data; Eq.: fitted curves).

The results of a typical fitted curve are compared with the experimental data in Figure 9.

The ultimate soil resistance (*p*_*u*_) was assumed to be related to the square of passive earth pressure coefficient (*k*_*p*_ = tan^2^(45° − *∅*/2)) [26]. Consider

(8) $$\begin{array}{c} \frac{p_{u}}{D}={Ak}_{p}^{2}\gamma^{\prime }z^{n}, \end{array}$$

where *γ*′ is effective unit weight of soil (KN/m^3^); *A*, *n* are curve-fitting constants; *z* is depth of soil (cm); and *D* is pile diameter (m). *P*_*u*_ was obtained from the *p*-*y* curves at each depth by fitting the experimental data points with a relationship of the form of [8]. Linear regression was used to obtain the best-fit values of the nondimensional parameters *A* and *n*. The average values of *A* were 0.093 and 0.062 and the average values of *n* were calculated 0.91 and 1.17 for dense and loose sand, respectively.

**Figure 10:**
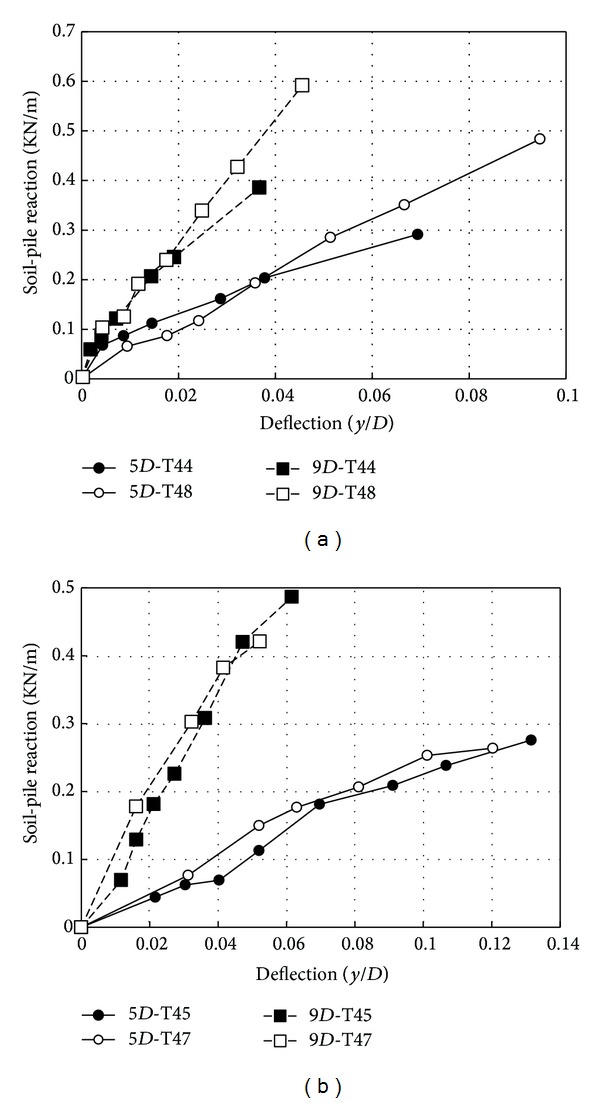
Experimental *p*-*y* curves of smooth and rough piles at different depths for Johor Bahru sand; (a) *D*_*r*_ = 75%; (b) *D*_*r*_ = 30%.

Load-transfer behavior is also a function of relative density of soil. A series of tests were conducted at different relative densities.  Figure 10 presents the experimental load-transfer curves for *D*_*r*_ = 75% and *D*_*r*_ = 30%. The initial subgrade modulus was increased with the increasing of depth (Figure 10). The different magnitude of *k*_ini_ for the different relative densities is shown in Figure 10. It is found that for a given load, decreasing the relative density causes an increase in the moments and deflections. It can be stated that the increase in deflection in smooth pile was more than that in rough pile.

**Figure 11:**
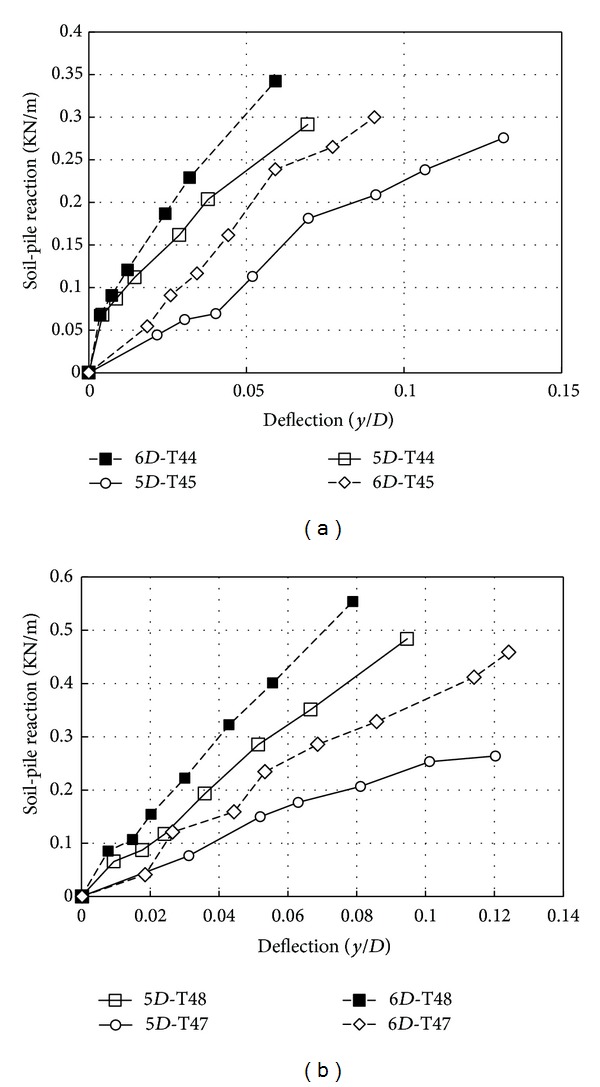
Experimental *p*-*y* curves in different relative densities with different depthsfor Johor Bahru sand; (a) smooth; (b) rough.

Figure 11 compares the results of experimental *p*-*y* curves obtained in loose and dense sand at 5*D* and 6*D* depths. It can be stated that, while there is similar trend in Figures 11(a) and 11(b), increasing the friction on the surface the pile had the significant influence on the soil-pile reaction. As Figures 11(a) and 11(b) show, the ratio of dense to loose density soil-pile reaction (*p*_dense_/*p*_loose_) ranges from 1.2 to 2.0 for the smooth pile at a small displacement and a ratio of 2.0 at a large displacement. For the rough pile, this ratio ranges from 2.0 to 3.0 at a small to large displacements. It is worth noting that this ratio decreased when lateral soil resistance increased with depth. However, in the smooth piles, the initial stiffness of *p*-*y* curves for dense sand was stiffer than that in loose sand (Figure 11).

### 4.1. Comparisons with Existing *p*-*y* Curves

**Figure 12:**
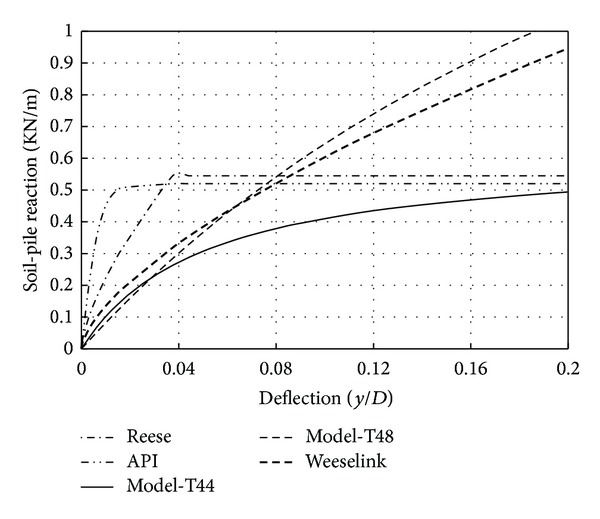
Comparison of *p*-*y* curves obtained with previous studies at the depth of 6*D.*

Practically, there are various *p*-*y* curves applied now in soil-pile interaction analysis. The procedures for generating *p*-*y* curves proposed by Reese et al. [15], Weeselink et al. [18], and O'Neill and Murchison [16] are widely used in professional jobs. The American Petroleum Institute (Ins.API) suggests the curve developed by O'Neill and Murchison [16]. The model proposed from this study is presented in Figure 12 and also compared with the three existing load-transfer curve models in dense sand at the depth of 6*D* (where *D* is pile diameter). Direct comparison of the *p*-*y* curve developed shows significant differences in the magnitude and shapes of the reaction-displacement response with the existing load-transfer curves. Initial stiffness of curves for the two silica sand models' Reese et al. [15]; O'Neill and Murchison [16] indicated a perfectly plastic behavior. The calcareous sand model that is presented by Weeselink et al. [18] and the *p*-*y* curves developed here show initial stiffness less than silica sand models. In other words, although the lateral pressure increased more gradually but the ultimate soil-pile reaction was larger than the *p*-*y* curves developed in dense sand.

**Figure 13:**
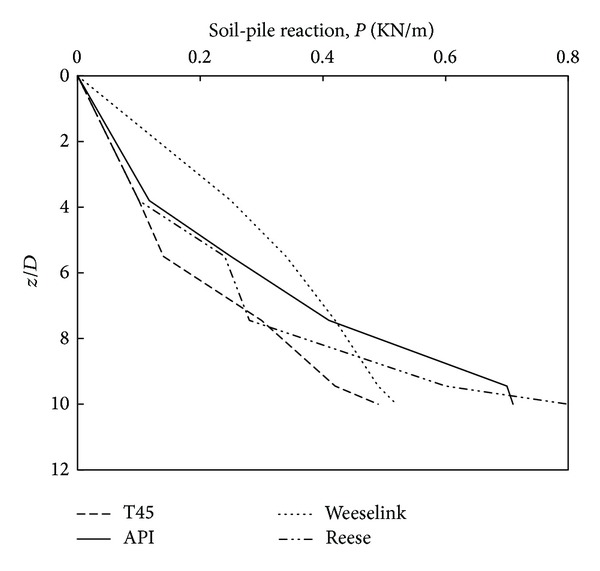
New profiles and existing soil-pile reaction for Johor sand at a strain of 5% (*y*/*D*).

Figure 13 illustrates a comparison of the soil-pile reaction from the proposed model with the three existing models at a normalized displacement of *y*/*D* = 0.05. It can be seen that the developed model, Reese's model and API's model, had the same pressure from the surface to depth of 4*D*. In additional, the proposed model, the API model, becomes more stiffer, and the Weeselink model becomes more softer with increasing the depth.

## 5. Conclusions

A series of model experiments have been conducted in sandy soil to determine the load-transfer (*p*-*y*) curve and pile behavior subjected to lateral load. The *p*-*y* curves were obtained using the strains recorded along the pile located in loose and dense sand. The experimental data were fitted by a hyperbolic function as well as several modification factors in order to consider the soil density and the wall friction of pile. Finally, the proposed *p*-*y* curves were compared with the existing *p*-*y* curves. The following conclusions are drawn based on this study.

1. The *p*-*y* curves developed show good agreement with the measurements.
2. The soil-pile reaction for various relative density (from 30% to 75%) was increased in range from 40 to 95% for smooth pile at a small displacement and 90% at a large displacement.
3. The nondimensional parameters in (8) and the average values of *A* were 0.093 and 0.062; and the average values of *n* were 0.91 and 1.17 for dense and loose sand, respectively.
4. The developed *p*-*y* curve shows a significant difference in the magnitude and shape compared with the existing load-transfer curves.
